# Circular RNA circRNA_100349 functions as a miR-218–5p sponge for suppressing the cell proliferation of gastric cancer via regulation of IGF2 expression

**DOI:** 10.1016/j.clinsp.2024.100492

**Published:** 2024-09-17

**Authors:** Linmei Lin, Jiamilan Wusiman, Zixu Zhang

**Affiliations:** a Blood Transfusion Department, The First Hospital of Putian City, Putian, Fujian, China; b Internal Medicine-Oncology, Guangzhou Royal Cancer Hospital, Guangzhou, Guangdong, China; c Department of Endoscope, Tongren Hospital Shanghai Jiao Tong University School of Medicine, Shanghai, China

**Keywords:** circRNA_100349, Gastric cancer, miR-218–5p, IGF2, Cell proliferation, circRNA

## Abstract

**Background:**

Circular RNAs (circRNAs) hold critical importance due to their notable function in developing Gastric Cancer (GC), which is a malignancy with the third most frequent occurrence worldwide. The aim of this study was to see if circRNA_0044516 would control GC cell proliferation and establish more effective therapeutic strategies.

**Methods:**

In GC tissues or cells, quantitative Real‑Time Polymerase Chain Reaction (qRT-PCR) was employed for the detection of the expression of circRNA_100349, Insulin-like Growth Factor II (IGF2), and miR-218–5p. CCK-8 assays were employed to gauge the proliferation of cells. A luciferase reporter was employed to establish the relationship of circRNA_100349 or IGF2 with miR-218–5p.

**Results:**

CircRNA_100349 was observed to undergo upregulation in GC cell lines along with tissues. GC cell proliferation was prevented by downregulating circRNA_100349. MiR-149 was targeted by CircRNA_100349, and its downregulation increased the amount of miR-218–5p in GC cells. Simultaneously silencing circRNA_100349 decreased IGF2 expression via miR-218–5p, and thus suppressed GC cell proliferation. Furthermore, in nude mice, circRNA_100349 knockdown prevented the tumor development of GC cells.

**Conclusions:**

The findings furnished evidence of the critical involvement of circRNA_100349 in GC and that its downregulation impedes GC cell proliferation via the miR-218–5p/IGF2 axis.

## Highlight

• CircRNA_100,349 is notably elevated and a high level of circRNA_100,349 predicts poor prognosis in GC.
• Downregulating circRNA_100,349 subdued proliferation of GC Cells via miR-218–5p/IGF2 axis.

## Introduction

The third most common cancer on the planet is gastric cancer.^1^^,^^2^ The vast majority of patients are generally diagnosed with advanced gastric cancer after the initial diagnosis due to a lack of apparent signs and screening services.^3^ Consequently, more research into the molecular-level mechanistic understanding of GC tumorigenesis and progression is urgently needed in order to establish further effective therapeutic regimens.

Circular RNAs (circRNAs) belong to a category of noncoding RNA that originates in introns, exons, or intergenic regions.^4^^,^^5^ Structurally, they exist as covalently closed-loop structures, unlike other linear noncoding RNAs.^6^ CircRNAs have been shown to affect a multitude of biological processes, for instance, oxidative stress, migration, cell proliferation, invasion, and immune responses.^7^^,^^8^ Furthermore, the expression of several circRNAs' expressions has been found to be dysregulated in a number of cancers according to recent studies. These include gastric cancer ^9^ breast cancer,^10^ ovarian cancer,^11^ colon cancer,^12^ non-small cell lung cancer,^13^ thereby implying that they may have a biological function in these cancers. CircRNAs were observed to be upregulated in GC tissues in comparison to non-GC tissues, as established by the GEO DataSets. circRNA_100,349, which is codified in chromosome 12, was chosen for further study among the upregulated circRNAs.

CircRNAs have the capacity to act as sponges for miRNAs to control a variety of diseases, GC being one of them.^14^, ^15^, ^16^ The authors discovered that miR-218–5p might be one of the miRNAs, that is a possible target of circRNA_100,349. This was discovered following bioinformatic research making use of circRNA and the miRNA prediction website (http://starbase.sysu.edu.cn/).^17^ As a result, the authors hypothesized that circRNA_100,349 regulates GC cell proliferation via regulation of the miR-218–5p/IGF2 pathway. The object of the current work was to ascertain the function and mechanism of circRNA_100,349 in GC cell proliferation regulation.

## Methods

### Patient tissue specimens

The authors acquired a total of 122 GC tissues along with the adjoining natural tissues from clinically diagnosed patients. Table 1 shows the clinical features of the individuals involved. Informed consent was obtained from all subjects and/or their legal guardian(s). The Ethics Committee of Tongren Hospital Shanghai Jiao Tong University School of Medicine (n° 2023–012) approved the present study.

**Table 1:** Correlation between clinical characteristic and circRNA_100,349 expression of patients with GC.

Clinical parameters	circRNA_100,349		p-values
	Low	High	
Age (years)			0.842
≥ 60	30	33	
< 60	31	28	
Sex			0.751
Male	27	29	
Female	34	32	
Tumor size (cm)			<0.05
≥ 5	49	15	
< 5	12	46	
TNM stage			<0.05
I‒II	47	11	
III‒IV	14	50	

### Cell lines and cell culture

BNBIO.COM provided GC cell lines of human origin namely SGC-7901, MNK-45, MGC-803, and HGC-27, as well as the immortalized human normal gastric mucosa cell line GES-1 (Beijing, China). The cells grew in RPMI-1640-based media that had 10 % fetal bovine serum (Thermo Fisher Scientific, USA) and 1 % penicillin/streptomycin (Thermo Fisher Scientific, USA) added to it. At 37 °C, the cells were incubated using 5 % CO_2_.

### Cell transfection

Using Lipofectamine 2000 Reagent, MKN-45 cells and MGC-803 cells were transfected using the miR-218–5p inhibitor, siRNA-circRNA 100,349, shRNA-circRNA_100,349, miR-218–5p mimic, siRNA-IGF2, along with negative controls (siRNA-NC, shRNA-NC, miR-NC mimic, anti-miR-NC, and siRNA-NC) (Invitrogen, USA).

### Quantitative real‑time polymerase chain reaction (qRT-PCR)

MTrizol Reagent was taken to remove complete RNA from GC cells and tissues (Invitrogen, USA). For analysis of circRNA_100,349, 1 mg RNA was used to synthesize cDNA making use of PrimeScript RT Reagent Kit (Takara, Japan) as directed by the supplier after being quantified by a spectrophotometer. The qRT-PCR analysis was then executed using the Step-One Plus Real-Time PCR Method (Applied Biosystems, USA) and SYBR Green I fluorescence kits (Takara, Japan). The 2^−△△CT^ methodology was employed for the calculation of the correlative gene expressions. Following is the sequence information of primers: circRNA_100,349: F5’CCATAGCACGGTCGCTGCAGG3’, R 5′AAAGCACAGAATCCCGCCACTACC3’; U6: F 5′CTCGCTTCGGCAGCACA3′, R5′CGCTTCACGAATTTGCGTGTCAT3′.

### Luciferase reporter assay

Two recombinant vectors (circRNA_100,349 WT and circRNA_100,349 Mut) were created by cloning the circRNA_100,349 or IGF2 bearing a Mutant (Mut) or Wild Type (WT) binding site of miR-218–5p within the pmirGLO plasmid (Promega, USA). Then, using Lipofectamine 2000 Reagent, 293T cells were subjected to co-transfection with circRNA_00349 WT or IGF2 WT and miR-NC mimic or miR-218–5p mimic, or with circRNA_100,349 Mut or IGF2 Mut and miR-218–5p mimic or miR-NC mimic obtained from Invitrogen, USA. The luciferase activity was then recorded by making use of the Dual-Luciferase Reporter Assay System (Promega, USA).

### CCK-8 assay

CCK-8 kits were taken for the detection of cell proliferation (Beyotime, China). 96-well plates (2 103 cells/well) were seeded respectively with 100 L MGC-803 and MKN-45 cell suspensions, following which 10 mL of CCK-8 solution was applied to individual wells. The cells were set up for incubation at 37 °C with 5 % CO_2_. Set at a wavelength equivalent to 450 nm, a microplate reader (Biorad, USA) was utilized to examine cellular proliferation.

### RNA immunoprecipitation

The RNA Immunoprecipitation (RIP) analysis was conducted following the instructions by the manufacturer using the Magna RIPTM RNA-binding protein immunoprecipitation kit (Millipore, USA). After transfection with miR-218–5p mimics or a negative regulation, MGC-803 and MKN-45 cells were allowed to lyse in full RNA immunoprecipitation lysis buffer. Using magnetic beads conjugated with anti-IgG or anti-Argonaute 2 antibodies (AGO2) (Millipore, USA), the cellular extract was then incubated for 6 hours at 4 °C. For the extraction of proteins, Proteinase K was used to wash and incubate the beads. Afterward, the TRIzol Reagent was utilized to remove the isolated RNA (Takara, Japan). The RNA after being purified was then run through an agarose gel electrophoresis followed by qRT-PCR analysis.

### Biotin-coupled probe RNA pull-down assay

Pull-down assays with biotinylated circRNA_100,349 and miR-218–5p (GenePharma, China) were performed as previously mentioned.^18^ After being harvested, 1 × 107 GC cells were lysed and later subjected to sonication. A two-hour incubation of the probe with probes-M280 streptavidin dynabeads (Invitrogen, USA) at 25 °C led to the formation of probe-coated beads. Using the probe-coated beads mixture, the cell lysates were allowed to incubate overnight at 4 °C The RNA complexes attached to the beads were eluted and Trizol Reagent was employed to purify them (Takara, Japan) upon washing with the wash buffer.

### In vivo assay

The Animal Care and Ethics Committee of Tongren Hospital Shanghai Jiao Tong University School of Medicine (n° 2022–039) approved the study, which was conducted in accordance with the National Institutes of Health's animal use guidelines and the ARRIVE guidelines. BALB/c nude mice were categorized into four groups of five mice, each at the age of four weeks. Subcutaneous injections of MGC-803 and MKN-45 cells stably infected with shRNA-circRNA_100,349 or shRNA-NC were given to each mouse in their right-side flanks to establish xenografts. Every seven days, the volume of tumors was estimated. The tumor masses were finally measured on day 28 after killing the mice.

### Elisa assay

MGC-803 and MKN-45 cells infected with siRNA-IGF2 or siRNA-NC culture supernatants had their IGF2 levels assessed. Apotech/Immunodiagnostic (San Diego, CA; complete sRANKL ELISA kit) and R&D Systems (human IGF2 ELISA kit) commercially available ELISA kits were used in the light of the manufacturer's directions A Anthos 2010 ELISA reader was employed to read the results at an optical density of 450 nm (Anthos Labtec Instruments Ges.m.b.H, Wals Salzburg, Austria).

### Statistical analysis

The mean and standard deviation of data from three individual experiments were calculated (SD). GraphPad Prism 7 software was used to compare groups using Student's *t*-test or one-way analysis of variance (ANOVA) followed by Tukey's test. Spearman rank correlation was utilized to perform correlation studies; p-values under 0.05 were deemed statistically important.

## Results

### CircRNA_100,349 is notably elevated and a high level of circRNA_100,349 predicts poor prognosis in GC

**Fig. 1:**
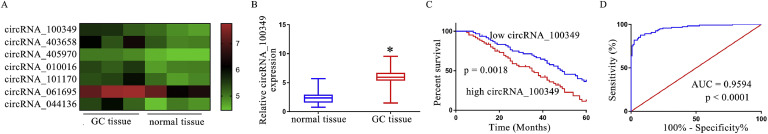
CircRNA_100,349 is notably elevated and a high level of circRNA_100,349 predicts poor prognosis in GC. (A) The substantially upregulated circRNAs in GC tissues in comparison to adjoining normal tissues is seen in a cluster heat map from GEO DataSets. CircRNA_100,349 was the most upregulated circRNA in GC tissues in comparison to adjoining normal tissues. (B) QRT-PCR based examination of the expression of circRNA_100,349 in GC. (C) Kaplan-Meier survival analysis was employed to determine the overall survival rate of individuals with GC who had high or low expression of circRNA_100,349. (D) Analysis of the sensitivity and specificity of circRNA_100,349 as a tumor biomarker for GC by ROC curve (* *p* < 0.05).

The substantially upregulated circRNAs in GC tissues relative to the normal adjoining tissues (*p* < 0.05) is evident in a cluster heat map from GEO DataSets (Fig. 1A). As a result, the authors concentrated on the most upregulated circRNAs and compared them to circBase. CircRNA_100,349, being the most upregulated circRNA was the prime object of our attention. In an attempt to confirm if there was an upregulation in the circRNA_100,349 expression level in GC tissues according to the GEO DataSets, the authors employed qRT-PCR to detect upregulation of circRNA_100,349 in 122 GC tissues when compared to the normal adjoining tissues (Fig. 1B), that existed in agreement with the GEO DataSets. The analysis of the clinicopathologic parameters manifested that circRNA_100,349 expression had a notable association with the TNM stage and size of the tumor in individuals with GC (Table 1). Additionally, in comparison to the individuals with elevated circRNA_100,349 expression, individuals with circRNA_100,349 under expression had a rather lower overall survival rate (Fig. 1C). Multivariate analysis of factors correlated with prognosis in patients with GC indicated that circRNA_100,349 level and TNM and tumor size were independent prognostic parameters of patients with GC (Table 2). Notably, circRNA_100,349 expression level has a prospective clinical value as a tumor biomarker according to the Receiver Operating Characteristic (ROC) curve (Fig. 1D, AUC = 0.9594). Collectively, these findings suggest that in GC, the level of circRNA_100,349 undergoes up-regulation, and poor prognosis of GC is apparently correlated with high circRNA_100,349 expressions.

**Table 2:** Multivariate analysis of prognostic factors in patients with GC.

Variable	Category	p-values
Age		0.753
	≥ 60	
	< 60	
Sex		0.851
	Male	
	Female	
Tumor size (cm)		<0.05
	≥ 5	
	< 5	
TNM stage		<0.05
	I‒II	
	III‒IV	
circRNA_100,349		<0.05
	Low	
	High	

### Knockdown of circRNA_100,349 subdued the proliferation of GC cells

**Fig. 2:**
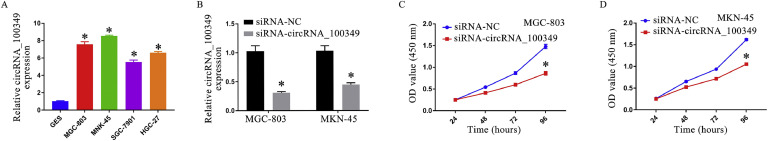
GC cell proliferation was prevented following the knock down of circRNA_100,349. (A) An analysis of circRNA_100,349 expressions in GC cells based on QRT-PCR. (B) qRT-PCR detected the expression of circRNA_100,349. (C and D) Cell proliferation of GC was determined by CCK-8 assay (* *p* < 0.05).

Finally, compared with the GES-1 cell line, the expression levels of circRNA_100,349 were verified in the SGC-7901, MNK-45, MGC-803, and HGC-27 cell lines (Fig. 2A). To probe into the functional influence of circRNA_100,349 on GC cells, the authors transfected the highest expression MGC-803 and MKN-45 cells with siRNA-circRNA_100,349, and the results showed revealed a successful knockdown circRNA_100,349 in GC cells (Fig. 2B). The findings of the CCK-8 assay manifested that siRNA-circRNA_100,349 transfected for 24–96 hours remarkably reduced GC cell proliferation (Fig. 2C and D). These findings indicated that knocking down circRNA_100,349 prevented GC cells from proliferating.

### MiR-218–5p is a target of circRNA_100,349 in GC cells

**Fig. 3:**
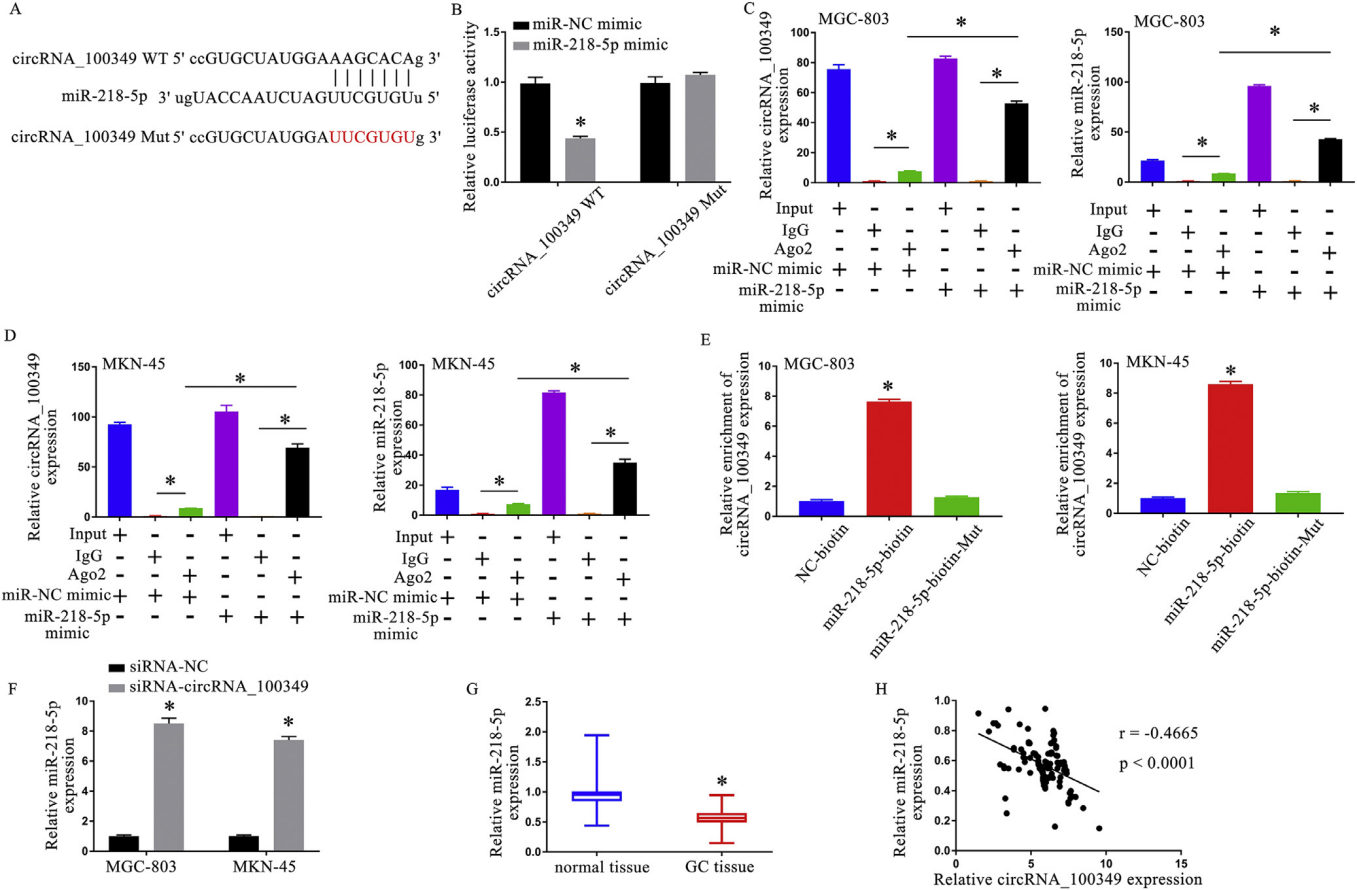
MiR-218–5p is a target of circRNA_100,349 in GC cells. (A) Starbase database predicted the binding site of miR-218–5p on circRNA_100,349. (B) The relative luciferase reporter activity. (C and D) Following transfection with mimics and NC, a RIP assay was performed in GC cells, followed by qRT-PCR to detect circRNA_100,349 and miR-218–5p. (E) RNA pull-down: The enrichment of circRNA_100,349 in the miR-218–5p pull-down complex was determined using qRT-PCR. (F) Inspection of miR-218–5p expression in GC cells based on QRT-PCR. (G) Inspection of miR-218–5p expression in GC based on QRT-PCR. H. Negative correlation was markedly showed between circRNA_100,349 and miR-218–5p (* *p* < 0.05).

The starbase database sequence of circRNA_100,349 (Fig. 3A), the luciferase reporter assay (Fig. 3B) and RIP analysis (Fig. 3C and D) revealed a direct relationship between circRNA_100,349 and miR-218–5p. In comparison to the miR-218–5p-biotin-Mut pull-down complexes, circRNA_100,349 was found to be more abundant in miR-218–5p-biotin pull-down complexes, according to RNA pull-down data (Fig. 3E). The knockdown of circRNA_100,349 subsequently led to a major increase in the concentration of miR-218–5p (Fig. 3F). Furthermore, a comparison of GC tissues with the adjacent normal tissues, revealed that miR-218–5p expression was indeed observed to be downregulated (Fig. 3G), circRNA_100,349 and miR-218–5p were seen to have a strong negative correlation (Fig. 3H). These findings showed that circRNA_100,349 targeted miR-218–5p and that its downregulation stimulated the expression of miR-218–5p.

### CircRNA_100,349 silence suppressed IGF2 expression through miR-218–5p

**Fig. 4:**
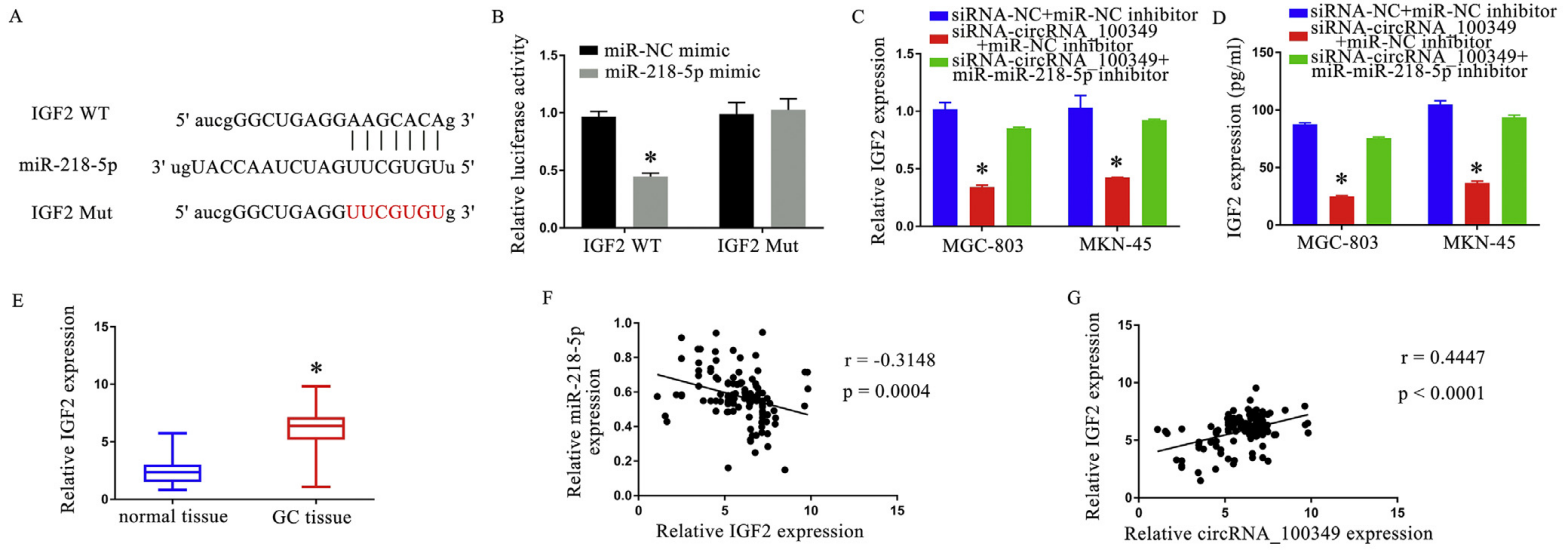
CircRNA_100,349 silence suppressed IGF2 expression via miR-218–5p. (A) The miR-218–5p-binding site on IGF2 was speculated by the TargetScan database. (B) The relative luciferase reporter activity. (C) Analysis of IGF2 expression in GC cells based on QRT-PCR. (D) Elisa assay of IGF2 expression in GC cells. (E) Analysis of IGF2 expression in GC based on QRT-PCR. (F) IGF2 and miR-218–5p were found to have a strong negative correlation. (G) Positive correlation was markedly showed between circRNA_100,349 and IGF2 (* *p* < 0.05).

The TargetScan database sequence of circRNA_100,349 (Fig. 4A) and the luciferase reporter assay (Fig. 4B) revealed a clear relation between IGF2 and miR-218–5p. IGF2 expression was reduced by siRNA-circRNA_100,349, though miR-218–5p inhibitor transfection reversed the patterns (Fig. 4C and D). Following that, in comparison to adjacent normal tissues, IGF2 expression was discovered to be upregulated in GC tissues (Fig. 4E). Furthermore, miR-218–5p expression in GC tissues was found to be negatively correlated with IGF2 expression (Fig. 4F) and positively correlated with circRNA_100,349 expressions (Fig. 4G). In total, the current findings showed that silencing circRNA_100,349 inhibited IGF2 expression via miR-218–5p.

### Downregulating circRNA_100,349 subdued proliferation of GC cells via miR-218–5p/IGF2 axis

**Fig. 5:**
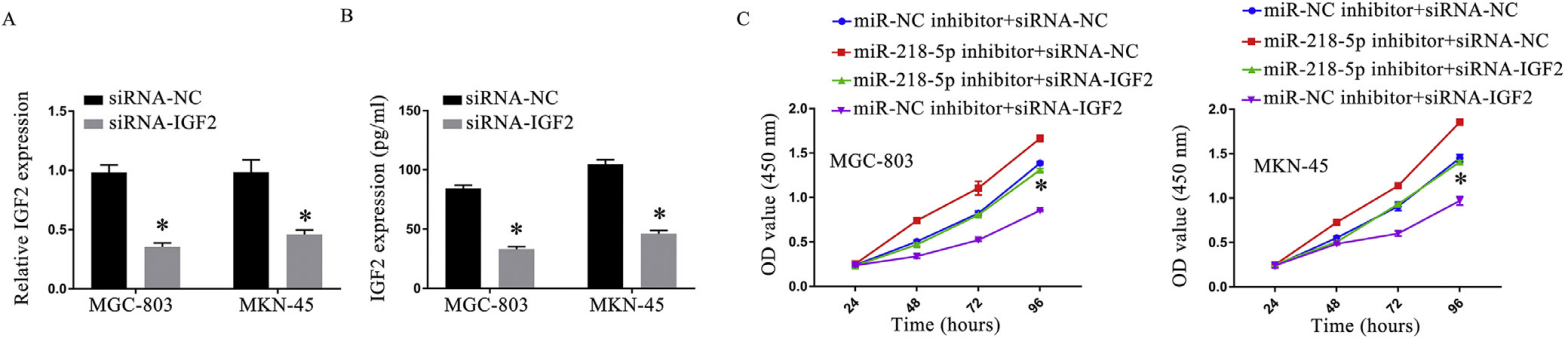
The miR-218–5p/IGF2 axis inhibited GC cell proliferation upon downregulation of circRNA_100,349. (A) QRT-PCR analysis of IGF2 expression in GC cells. (B) Elisa assay of IGF2 expression in GC cells. (C) Cell proliferation of GC was ascertained by CCK-8 assay (* *p* < 0.05).

To clarify if circRNA_00349 modulates GC cell proliferation through the miR-218–5p/IGF2 axis, cell proliferation of GC cells was measured following transfecting with a miR-218–5p inhibitor or siRNA-IGF2. In GC cells, transfection with siRNA-IGF2 reduced IGF2 expression (Fig. 5A and B). Overexpressing IGF2 countermand the impact of circRNA_100,349 silences on cell proliferation, and knocking down miR-218–5p abrogated the suppression of cellular proliferation caused by transfection with miR-218–5p inhibitor (Fig. 5C). Downregulating circRNA_100,349 inhibited GC cell proliferation through the miR-218–5p/IGF2 axis, according to these findings.

### Knockdown of circRNA_100,349 resulted in attenuation of tumor growth in nude mice

**Fig. 6:**
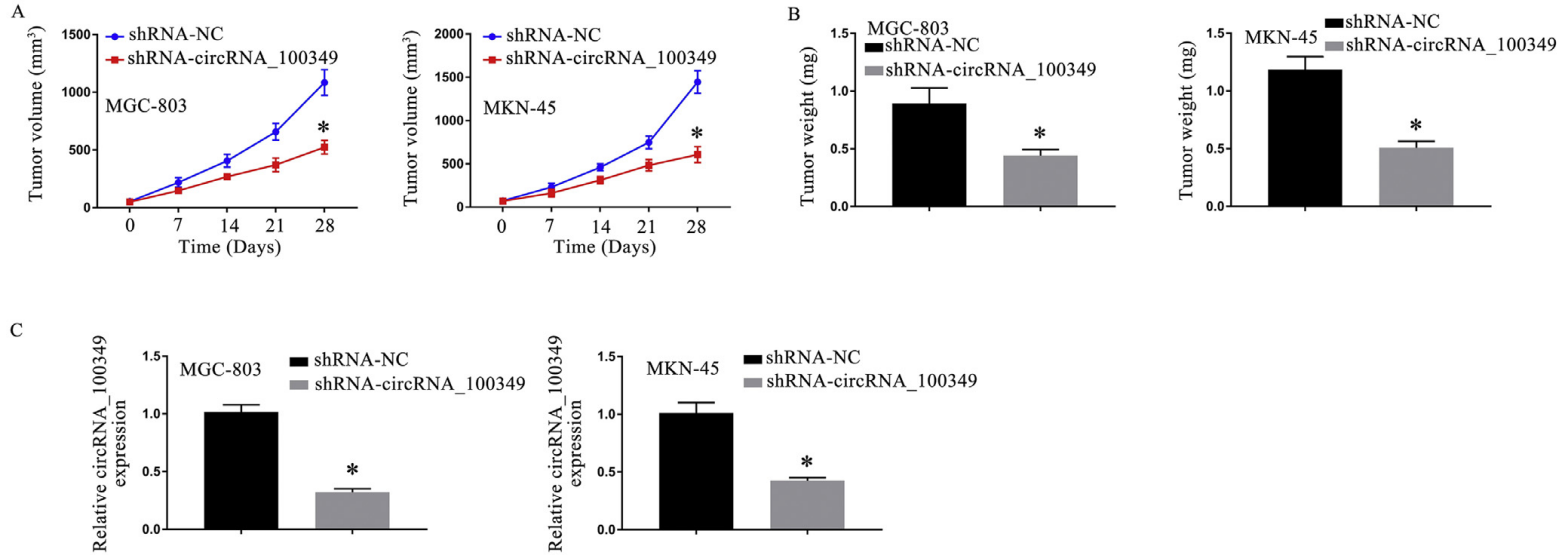
In nude mice, tumor growth was attenuated by the knockdown of circRNA_100,349. The tumor volume (A) and weight (B) of tumors. (C) qRT-PCR detected the expressions of circRNA_100,349 (* *p* < 0.05).

The authors used a nude mouse xenograft tumor model to verify the action of circRNA_100,349 in GC. The knockdown of circRNA_100,349 inhibited tumor development, (Fig. 6A) and decreased tumor weight (Fig. 6B) in MGC-803 and MKN-45 cells according to the findings. Besides, qRT-PCR assay showed circRNA_100,349 knockdown decreased circRNA_100,349 expressions in excised tumors (Fig. 6C).

## Discussion

The present investigation focused on the mechanism and purpose of circRNA_100,349 in controlling the proliferation of GC cells in order to fill in the existing research gaps regarding the involvement of circRNA_100,349 in GC. Initially, the authors demonstrated the upregulation of circRNA_100,349 in GC cells and tissues. Following that, the authors discovered that knocking out circRNA_100,349 subsequently led to a decrease in GC cell proliferation. Additionally, the authors confirmed that miR-218–5p acts as a target miRNA of circRNA_100,349 and IGF2. Finally, it was established that the downregulation of circRNA_100,349 regulated the miR-218–5p/IGF2 axis and resultantly blocked the proliferation of GC cells.

CircRNAs belong to a category of noncoding RNA that originates in introns, exons, or intergenic regions.^4^^,^^5^ CircRNA can serve as an excellent biomarker in tumors, such as circRNA CDR1as in GC,^19^ circSLIT2 in GC,^20^ and circYPEL2 in cervical cancer.^21^ Additionally, circRNA can regulate tumor progression via the circRNA/miRNA/mRNA axis. For example, hsa_circRNA_104,348 promotes hepatocellular carcinoma progression by modulating miR-187–3p/RTKN2 axis and activating Wnt/β-catenin pathway.^22^ The interactions between circRNA and RNA Binding Proteins (RBPs) are also considered one of the functions of circRNA. For example, circRHOBTB3 represses metastasis by regulating the HuR-mediated mRNA stability of PTBP1 in colorectal cancer.^23^ Notably, circRNAs have been shown to possess regulatory characters in GC in a growing number of studies. Most circRNAs are dysregulated in GC, as demonstrated by previous research. The miR-149–5p/YWHAZ axis, for example, has a special function in the progression of gastric cancer.^24^ As for gastric cancer, CircRNA_104,433 targets miR-497–5p, thereby controlling cell growth.^25^ By controlling the Wnt/-catenin pathway, Cir-ITCH prevents gastric cancer migration, invasion, and proliferation.^26^ In the current work, circRNA_100,349 was discovered to have an increased expression in GC tissues and cells. Additional investigation regarding the function of circRNA_100,349 in GC was thus carried out. According to the findings, downregulating circRNA_100,349 inhibited GC cell proliferation. Thus, these findings show that circRNA_100,349 works like an oncogene in GC cells, promoting cell proliferation and that its downregulation might be a significant therapeutic area in GC for cell proliferation inhibition. While this study established the part played by circRNA_100,349 in the proliferation of GC cells, further research is needed to ascertain the involvement of several other dysregulated circRNAs in GC.

CircRNAs are thought to control how various target genes are expressed and they do so by sponging miRNAs, according to a number of earlier works. For example, the circular RNA MTO1 slows the progression of gastric cancer by increasing Prostate Apoptosis Response-4 protein (PAWR) levels by sponging miR-199a-3p.^27^ In gastric cancer, the circular RNA MCTP2 impedes cisplatin resistance by inducing Myotubularin Related protein 3 (MTMR3) expression via miR-99a-5p.^28^ A miR-218–5p-binding site was discovered on circRNA_100,349 in the work based on bioinformatics research, suggesting that circRNA_100,349 may well operate by targeting miR-218–5p, which has been observed to play critical roles in GC.^29^ The present findings showed that circRNA_100,349 could target miR-218–5p, and that downregulating circRNA_100,349 increased miR-218–5p expression. These discoveries showed that circRNA_100,349 specifically targeted miR-218–5p and suppressed miR-218–5p expression. findings also verified that downregulating circRNA_100,349 in GC cells decreased IGF2 expression, while miR-218–5p inhibitor increased it. Overall, the current findings persuaded us that circRNA_100,349 influenced IGF2 expression in GC by sponging miR-218–5p.

IGF2 has been identified as a critical regulatory molecule in cell proliferation regulation. By interfering with proliferation and survival signaling, miR-100 suppresses IGF2 and prevents breast tumorigenesis.^30^ NSCLC tumor growth and metastasis are inhibited by MicroRNA-615–3p, which inhibits IGF2.^31^ In this analysis, circRNA_100,349 downregulation inhibited GC cell proliferation, while miR-218–5p inhibitor or siRNA-IGF2 had an opposing impact. As a result, these findings showed that circRNA_100,349 silence inhibited GC cell proliferation through the miR-218–5p/IGF2 axis.

Finally, the present findings revealed that circRNA_100,349 was abundantly expressed in GC tissues and cells. The miR-218–5p/IGF2 axis inhibited the proliferation of GC cells following circRNA_100,349 knockdown. This discovery could lead to an expansion in the understanding of inhibiting the proliferation of GC cells.

## Availability of data and materials

The datasets used and/or analyzed during the current study are available from the corresponding author on reasonable request.

## Authors’ contributions

Linmei Lin and Zixu Zhang made substantial contributions to the conception and design of the study. Linmei Lin and Jiamilan Wusiman performed the data analysis and interpretation. Linmei Lin drafted the manuscript. Zixu Zhang performed the critical revision of the manuscript. All co-authors have read the manuscript and approved the final version for submission and publication to this journal.

## Declaration of competing interest

The authors declare no conflicts of interest.

## References

[1] Bray F., Ferlay J., Soerjomataram I., Siegel R.L., Torre L.A., Jemal A. (2018). Global cancer statistics 2018: GLOBOCAN estimates of incidence and mortality worldwide for 36 cancers in 185 countries. CA Cancer J Clin.

[2] Siegel R.L., Miller K.D., Jemal A. (2020). Cancer statistics, 2020. CA Cancer J Clin.

[3] Maconi G., Manes G., Porro G.B. (2008). Role of symptoms in diagnosis and outcome of gastric cancer. World J Gastroenterol.

[4] Li X., Yang L., Chen L.L. (2018). The biogenesis, functions, and challenges of circular RNAs. Mol Cell.

[5] Memczak S., Jens M., Elefsinioti A., Torti F., Krueger J., Rybak A. (2013). Circular RNAs are a large class of animal RNAs with regulatory potency. Nature.

[6] Qu S., Yang X., Li X., Wang J., Gao Y., Shang R. (2015). Circular RNA: a new star of noncoding RNAs. Cancer Lett.

[7] Panda A.C., Abdelmohsen K., Gorospe M. (2017). SASP regulation by noncoding RNA. Mech Ageing Dev.

[8] Hombach S., Kretz M. (2016). Non-coding RNAs: classification, biology and functioning. Adv Exp Med Biol.

[9] Yu X., Xiao W., Song H., Jin Y., Xu J., Liu X (2020). CircRNA_100876 sponges miR-136 to promote proliferation and metastasis of gastric cancer by upregulating MIEN1 expression. Gene.

[10] Xing L., Yang R., Wang X., Zheng X., Yang X., Zhang L. (2020). The circRNA circIFI30 promotes progression of triple-negative breast cancer and correlates with prognosis. Aging (Albany NY).

[11] Zhao Y., Hu Y., Shen Q., Chen Q., Zhu X.J., Jiang S.S. (2020). CircRNA_MYLK promotes malignant progression of ovarian cancer through regulating microRNA-652. Eur Rev Med Pharmacol Sci.

[12] Zhou P., Xie W., Huang H.L., Huang R.Q., Tian C., Zhu H.B. (2020). circRNA_100859 functions as an oncogene in colon cancer by sponging the miR-217-HIF-1alpha pathway. Aging (Albany NY).

[13] Yuan J., Song Y., Pan W., Li Y., Xu Y., Xie M. (2020). LncRNA SLC26A4-AS1 suppresses the MRN complex-mediated DNA repair signaling and thyroid cancer metastasis by destabilizing DDX5. Oncogene.

[14] Xin D., Xin Z. (2020). CircRNA_100782 promotes roliferation and metastasis of gastric cancer by downregulating tumor suppressor gene Rb by adsorbing miR-574-3p in a sponge form. Eur Rev Med Pharmacol Sci.

[15] Peng L., Sang H., Wei S., Li Y., Jin D., Zhu X. (2020). circCUL2 regulates gastric cancer malignant transformation and cisplatin resistance by modulating autophagy activation via miR-142-3p/ROCK2. Mol Cancer.

[16] Wu J., Chen Z., Song Y., Zhu Y., Dou G., Shen X. (2020). CircRNA_0005075 suppresses carcinogenesis via regulating miR-431/p53/epithelial-mesenchymal transition axis in gastric cancer. Cell Biochem Funct.

[17] Li J.H., Liu S., Zhou H., Qu L.H., Yang J.H. (2014). starBase v2.0: decoding miRNA-ceRNA, miRNA-ncRNA and protein-RNA interaction networks from large-scale CLIP-Seq data. Nucleic Acids Res.

[18] Du W.W., Yang W., Liu E., Yang Z., Dhaliwal P., Yang B.B. (2016). Foxo3 circular RNA retards cell cycle progression via forming ternary complexes with p21 and CDK2. Nucleic Acids Res.

[19] Li R., Tian X., Jiang J., Qian H., Shen H., Xu W. (2023). CircRNA CDR1as: a novel diagnostic and prognostic biomarker for gastric cancer. Biomarkers.

[20] Wang L., Xiao S., Zheng Y., Gao Z. (2023). CircRNA circSLIT2 is a novel diagnostic and prognostic biomarker for gastric cancer. Wien Klin Wochenschr.

[21] Zhang X., Yang S., Chen W., Dong X., Zhang R., Ye H. (2021). Circular RNA circYPEL2: a novel biomarker in cervical cancer. Genes (Basel).

[22] Huang G., Liang M., Liu H., Huang J., Li P., Wang C. (2020). CircRNA hsa_circRNA_104348 promotes hepatocellular carcinoma progression through modulating miR-187-3p/RTKN2 axis and activating Wnt/beta-catenin pathway. Cell Death Dis.

[23] Chen J., Wu Y., Luo X., Jin D., Zhou W., Ju Z. (2021). Circular RNA circRHOBTB3 represses metastasis by regulating the HuR-mediated mRNA stability of PTBP1 in colorectal cancer. Theranostics.

[24] Hui C., Tian L., He X. (2020). Circular RNA circNHSL1 Contributes to Gastric Cancer Progression Through the miR-149-5p/YWHAZ Axis. Cancer Manag Res.

[25] Wei W., Mo X., Yan L., Huang M., Yang Y., Jin Q. (2020). Circular RNA profiling reveals that circRNA_104433 regulates cell growth by targeting miR-497-5p in Gastric Cancer. Cancer Manag Res.

[26] Peng Y., Wang H.H. (2020). Cir-ITCH inhibits gastric cancer migration, invasion and proliferation by regulating the Wnt/beta-catenin pathway. Sci Rep.

[27] Song R., Li Y., Hao W., Yang L., Chen B., Zhao Y. (2020). Circular RNA MTO1 inhibits gastric cancer progression by elevating PAWR via sponging miR-199a-3p. Cell Cycle.

[28] Sun G., Li Z., He Z., Wang W., Wang S., Zhang X. (2020). Circular RNA MCTP2 inhibits cisplatin resistance in gastric cancer by miR-99a-5p-mediated induction of MTMR3 expression. J Exp Clin Cancer Res.

[29] Deng M., Zeng C., Lu X., He X., Zhang R., Qiu Q. (2017). miR-218 suppresses gastric cancer cell cycle progression through the CDK6/Cyclin D1/E2F1 axis in a feedback loop. Cancer Lett.

[30] Gebeshuber C.A., Martinez J. (2013). miR-100 suppresses IGF2 and inhibits breast tumorigenesis by interfering with proliferation and survival signaling. Oncogene.

[31] Liu J., Jia Y., Jia L., Li T., Yang L., Zhang G. (2019). MicroRNA 615-3p inhibits the tumor growth and metastasis of NSCLC via inhibiting IGF2. Oncol Res.

